# The Effect of Intratympanic Dexamethasone with Oral Prednisolone as a Primary Treatment in Idiopathic Sudden Sensorineural Hearing Loss 

**Published:** 2012

**Authors:** Mohammad Taghi Khorsandi Ashtiani, Pedram Borgheie, Nasrin Yazdani, Shirin Maghsoud

**Affiliations:** 1 *Department of otorhinolaryngology, Amir-Alam Hospital, Tehran University of Medical Sciences, Tehran, Iran*

**Keywords:** Dexamethasone, Emergency, Oral, Otolaryngologic, Prednisolone, Pure tone audiometry, Sensorineural hearing loss, Speech discrimination test, Speech reception threshold test.

## Abstract

**Introduction:**

Sudden sensorineural hearing loss (SSNHL) is a true emergency that must be diagnosed and treated immediately. The purpose of this study is to compare the efficacy of treatment with intratympanic dexamethasone plus oral prednisolone daily or every other day with that of treatment with oral prednisolone alone.

**Materials and Methods:**

Sixty-three patients with SSNHL that had been present for less than 10 days prior to the start of treatment were randomly allocated to three different groups. Patients in group A were treated daily with oral prednisolone 1 mg/kg for 10 days plus intratympanic dexamethasone 2 mg for the first 3 days of treatment. Patients in group B were treated every other day with oral prednisolone 1 mg/kg for 10 days with the addition of intratympanic dexamethasone 2 mg for the first 3 treatments. Patients in group C were treated daily with oral prednisolone 1 mg/kg alone for 10 days. Audiometric parameters including pure tone audiometry (PTA), speech reception threshold (SRT), and speech discrimination score (SDS) were assessed on days 1,5, and 10.

**Results:**

There was a significant improvement in PTA, SRT and SDS in each group over the 10 days but the greatest improvement was seen in the SRT measurements of group A in comparison with group B (19.81 ± 2.15, P=0.04) and C (26.26 ± 0.08, P=0.01). The difference in SRT between groups B and C was not statistically significant.

**Conclusion:**

The administration of intratympanic dexamethasone 2 mg daily for 3 days has an additive effect to that of 10 days of oral prednisolone 1 mg/kg in the treatment of SSNHL.

## Introduction

Sudden sensorineural hearing loss (SSNHL) is commonly encountered in audiologic and otolaryngologic practice. SSNHL is most commonly defined as sensorineural hearing loss of 30 dB or greater over at least three contiguous audiometric frequencies occurring within a 72-hr period. Steroid therapy is the current mainstay of treatment of idiopathic SSNHL (1) and systemic steroids produce significant hearing improvements for patients with moderate to severe SSNHL (2). Recently, many patients’ symptoms have been managed with intratympanic steroid therapy but no satisfactory comparative effectiveness study to support this practice exists. 

## Materials and Methods

Patients with idiopathic unilateral SSNHL who were referred to our hospital during the first 10 days following the onset of symptoms were included in this study. After obtaining written informed consent from the patients they were divided into three groups. Patients in group A were treated with oral prednisolone 1 mg/kg every day for 10 days plus intratympanic dexamethasone 2 mg for the first 3 days. Patients in group B were treated with oral prednisolone 1mg/kg every other day for 10 days with the addition of intratympanic dexamethasone 2 mg for the first 3 treatments. Patients in group C were treated with oral prednisolone 1 mg/kg alone for 10 days. 

For the intratympanic steroid injection topical anesthesia was applied with a 10% lidocaine spray from a spray pump. With the patient in the supine position and with the head tilted 45 degrees towards the opposite side, a 25-gauge spinal needle was passed through the anterosuperior portion of the tympanic membrane. Approximately 0.5 mL of dexamethasone (4 mg/mL) was then injected. Audiometry was performed on following the first systemic steroid days 1,

5, and 10 treatment. Statistical analysis of the differences between the measurements made in each patient group was performed using an ANOVA for comparison between groups and Student’s t-test for continuous variables (SPSS ver. 14.0, SPSS Inc). All patients underwent MRI as a part of their diagnostic work up to rule out retrochoclear lesions. 

## Results

A total of 63 patients were enrolled in the study but 18 dropped out during follow up due to non-medical reasons (7, 6, and 5 patients in group A, B, and C, respectively). Therefore, at the completion of the study 45 patients still remained, which included 14 patients in group A, 15 patients in group B, and 16 patients in group C. Of the patients 28 were women and 17 were men and the study was carried out on 24 left and 21 right ears. The mean age of the patients was 50±16 years (range: 20–70). 

The results of the MRI showed no retrocochlear lesions in any patients. Tables 1, 2, and 3 show the audiometric profiles of the different groups. Table 4 shows comparisons of the changes in the audiometric profile of patients between groups during treatment.

Most of the patients were middle-aged women whose left ears were most commonly affected. All the groups of patients showed improvement in their audiologic profiles with treatment. The mean increase in PTA in patients in groups A, B, and C was 41, 28, and 25 dB, respectively (*P*< 0.05), the mean decrease in SRT was 52, 32, and 25 dB, respectively (*P*<0.05), and the mean increase in SDS was 19, 11, and 18, respectively (*P*<0.05). By comparing between the patient groups we can see that the improvement in PTA in group A was 13 dB more than that observed in group B and 16 dB more than that in group C, although this difference was not statistically significant. The improvement in SRT in group A was significantly more than in groups B and C (20 and 27dB, respectively). There was no significant difference between any of the measurements in groups B and C.

**Table 1:** Changes in pure tone audiometry (PTA) on days 1, 5, and 10 of treatment Data are presented as mean ± SD

**Patient Group**	**PTA Day 1**	**PTA Day 5**	**PTA Day 10**	**PTA Day 1 – Day 10**	*P*
A	55.00 ± 8.38	57.00 ± 7.19	13.57 ± 4.37	41.42 ± 4.01	0.00
B	60.33 ± 9.43	49.66 ± 8.34	34.70 ±10.45	28.33 ± 1.02	0.00
C	60.47 ± 7.26	55.88 ± 13.93	34.58 ± 13.18	25.88 ± 5.09	0.01
					

**Table 2:** Changes in speech reception threshold (SRT) on days 1, 5, and 10 of treatment Data are presented as mean ± SD

**Patient Group**	**SRT Day 1**	**SRT Day 5**	**SRT Day 10**	**SRT Day 1 - Day 10**	*P*
A	17.09 ± 65.71	41.84 ± 15.97	12.45 ± 14.60	4.64 ± 52.14	0.00
B	12.55 ± 70.66	56.00± 7.07	5.76 ± 38.33	6.79 ± 32.33	0.00
C	10.29 ± 66.76	6.70 ± 55.88	5.73 ± 40.58	4.56 ± 25.88	0.01

**Table 3:** Changes in speech discrimination score (SDS) on days 1, 5, and 10 of treatment Data are presented as mean ± SD

		**SDS Day 1**	**SDS Day 5**	**SDS Day 10**	**SDS Day 10 – Day 1**	**P **
**A**	79.33 ± 18.77	93.57 ± 10.28	98.85 ± 8.86	19.33 ± 9.91	0.00
**B**	80.64 ± 10.42	69.09 ± 14.77	92.72 ± 9.85	11.01 ± 0.98	0.01
**C**	72.76 ± 8.50	79.53 ± 8.50	90.58 ± 5.23	18.30 ± 3.50	0.00

**Table 4:** Comparison between patient groups of mean differences in changes in PTA, SRT, and SDS after 10 days of treatment

	**Group A vs. B**	**Group A vs. C**	**Group B vs. C**
Changes in PTA	13.09; *P*=0.08	15.54; *P*= 0.06	2.45; *P*= 0.63
Changes in SRT	19.81; *P*= 0.04	26.26; *P*= 0.01	6.45; *P*= 0.82
Changes in SDS	8.97; *P*= 0.11	6.41; *P*= 0.85	7.29; *P*= 0.13

## Discussion

It seems that the addition of intratympanic dexamethasone to a daily oral steroid for 3 consecutive days can improve the hearing profile of patients with SSNHL in comparison with an oral steroid alone. These results are consistent with most of the previous studies in this field. For example, previous studies have shown that intratympanic dexamethasone improves hearing levels in diabetic patients by approximately 10 dB after the occurrence of SSNHL (3). Mattox and colleagues used an oral steroid together with intratympanic dexamethasone for the treatment of SSNHL and showed a significant improvement in hearing at 250 HTZ, although the overall rate of improvement was not significant when compared to oral prednisolone alone; 73% and 70% respectively (4). In contrast, Battaglia and colleagues compared 3 different methods of steroid treatment and concluded that combination therapy is more beneficial in comparison with intratympanic dexamethasone or oral prednisolone alone (5). Rauch and colleagues compared the use of oral prednisone for 14 days with a 5-day taper and 4 doses over 14 days of methylprednisolone 40 mg/mL injected into the middle ear. The results of study showed an improvement in PTA by 30.7 dB following oral prednisone compared with an improvement of 28.7 dB in the intratympanic treatment group. Measurements of hearing level 2 months after treatment also showed that intratympanic treatment produced similar results to treatment with oral prednisone and therefore was not inferior (6). Spear and colleagues performed a review of studies that considered intratympanic steroid treatment for SSNHL and concluded that the use of an intratympanic steroid as the primary treatment for SSNHL appears equivalent to treatment with high-dose oral prednisone (7). Park and colleagues compared the efficacy of simultaneous and subsequent intratympanic dexamethasone injections for the treatment of idiopathic SSNHL and found that simultaneous intratympanic dexamethasone did not confer any additional hearing gains or an earlier recovery rate compared with subsequent intratympanic dexamethasone so they recommended that intratympanic dexamethasone is used only for subsequent or salvage treatment of idiopathic SSNHL after systemic steroid treatment (8). 

## Conclusion

The results of our study are consistent with the previous mentioned studies and are in favor of intratympanic dexamethasone addition to oral steroid for treatment of SSNHL. Further studies are recommended to assess the benefit of different dosage of intratympanic dexamethasone in SSNHL treatment.
